# Identifying defective solar cells in electroluminescence images using deep feature representations

**DOI:** 10.7717/peerj-cs.992

**Published:** 2022-05-19

**Authors:** Alaa S. Al‐Waisy, Dheyaa Ahmed Ibrahim, Dilovan Asaad Zebari, Shumoos Hammadi, Hussam Mohammed, Mazin Abed Mohammed, Robertas Damaševičius

**Affiliations:** 1 Computer Engineering Techniques Department, Information Technology College, Imam Ja’afar Al-Sadiq University, Baghdad, Iraq; 2 Department of Computer Science, College of Science, Nawroz University, Duhok, Kurdistan Region, Iraq; 3 Computer Science Department, Al-Ma’aref University College, Ramadi, Anbar, Iraq; 4 Computer Center, University of Anbar, Ramadi, Anbar, Iraq; 5 Information systems Department, College of Computer Science and Information Technology, University of Anbar, Ramadi, Anbar, Iraq; 6 Department of Software Engineering, Kaunas University of Technology, Kaunas, Lithuania

**Keywords:** Electroluminescence imaging, Solar cells, Photovoltaics, Defect recognition, Deep learning

## Abstract

Electroluminescence (EL) imaging is a technique for acquiring images of photovoltaic (PV) modules and examining them for surface defects. Analysis of EL images has been manually performed by visual inspection of images by experts. This manual procedure is tedious, time-consuming, subjective, and requires deep expert knowledge. In this work, a hybrid and fully-automated classification system is developed for detecting different types of defects in EL images. The system fuses the deep feature representations extracted from two different deep learning models (Inception-V3 and ResNet50) to form more discriminative feature vectors. These feature vectors are then fed into the classifier layer to assign them into one of different types of defects. A large-scale, challenging solar cells dataset composed of 2,624 EL images was used to assess the performance of the proposed system in both the binary classification (functional vs defective) task and multi-class classification (functional, mild, moderate, and severe) task. The proposed system has managed to detect the correct defect type with less than 1 s per image with an accuracy rate of 98.15% and 95.35% in the binary classification and multi-classification task, respectively.

## Introduction

People and governments are taking steps to decrease consumption of fossil fuels used in transportation and power plants and increase the use of green (renewable) energy sources (Okewu et al., 2017). Solar energy systems are a renewable energy source and have gained wide attention in recent years, especially in microgrids (Ayodele et al., 2019). Solar power plants have been developed around the world, resulting in the activation of large-scale manufacturing facilities that create solar energy components (Demirci, Beşli & Gümüşçü, 2021). One of the most important and sensitive components is the solar panels which should be protected from damage. Solar cell damage is primarily caused by environmental exposure or during the manufacturing process of solar panels. Solar panels are often shielded from environmental impacts such as rain, wind, and snow by an aluminum frame and a layer of glass lamination on the outside. Although these safeguards are in place, they are not always effective in preventing mechanical damages such as when the PV module is dropped during installation, the effect of falling tree limbs, hail, or heat stress. Apart from that, manufacturing defects such as improper soldering or defective wires are also able to result in damaged PV modules. Such faults have a negative impact on solar module quality, increase the amount of electricity lost, and considerably decrease the efficiency of solar panels (Li et al., 2019; Deitsch et al., 2019). Therefore, the solar cell should be used carefully to avoid damaged that might affect its performance.

The electroluminescence (EL) imaging is a technique that provide an images of photovoltaic (PV) modules and examining them to provide insights into a range of some defects on the surface of PV modules (*i.e*. damaged cells) (Buerhop et al., 2018). Consequently, it is vital to monitor the state of solar modules and to replace or repair any units that are found to be defective to ensure that solar power plants operate at their greatest efficiency (Akram et al., 2019). The EL image examination manually is an expensive and time-consuming task. It requires sufficient experience and knowledge of the subject, and is only possible on a small scale. Autonomous PV inspection is important when working on a wide scale (Bedrich et al., 2018; Bedrich et al., 2018).

Therefore, the main objective of this work is to propose an automated trainable model that can help to identify different defects of solar cell using the advance deep learning techniques. To achieve, the goal, a hybrid and fully automated supervised classification system for the automated detection of different defects in EL images of solar cells is developed. The proposed classification system depends on the feature representations extracted from two deep learning approaches (Inception-V3 and ResNet50). The contributions of this study can be summarized as follows.

- A hybrid and fully automated supervised classification system for automated identification of different faults in EL images of solar cells is developed. This is the first work to develop such a solution for distinguishing between EL images of functional and defective solar cells using powerful and discriminative feature representations obtained from two deep learning models (Inception-V3 and ResNet50).
- A training strategy supported by training tricks (dropout method, data augmentation) is employed to avoid overfitting and enhance generalization capability of deep learning models.

The remainder of this paper is structured as follows: “Related Work” includes briefly review for the current state-of-the-art related works. “Proposed System” provides an overview of the proposed system. The experimental results of the proposed system are presented in “Experimental Results”. Finally, the conclusions and future research directions are reported in the “Conclusions and Future Work”.

## Related work

Recently, visual inspection and assessment of solar modules using EL imaging techniques is an important topic of research. Most relevant studies have concentrated on the identification of the intrinsic/extrinsic defects, while less attention has been paid to the identification of the defects that can significantly reduce the efficiency of solar modules. In general, computerized crack identification methods can be separated into three different categories: spatial-based methods, spectral-based methods, and classification-based methods. Spatial-based methods can identify the crack in an image by detecting the variation in intensity between the background of the image and crack area (Hoang, 2018; Xu et al., 2018). An example on the classification-based methods was proposed by Acharya, Sahu & Jena (2019) and other researchers (Buerhop-Lutz et al., 2018; Mathias et al., 2020; Wang et al., 2021).

In most cases, they were classifying the EL image into only a limited type of defects. The performance of the most works was reported using extremely small dataset, which can not reveal the real performance of the proposed approaches. Furthermore, some works are suffering from detecting the cracks in low-intensity images and computationally demanding. Thus, a hybrid and fully automated classification system based on different CNN architectures for identifying different types of defects in EL images of solar cells are developed using large-scale and challenging EL images dataset. To the best of our knowledge, this is the first attempt to distinguish between EL images of functional and defective solar cells using powerful and discriminative feature representations extracted from two different deep learning descriptors.

## Proposed system

**Figure 1:**
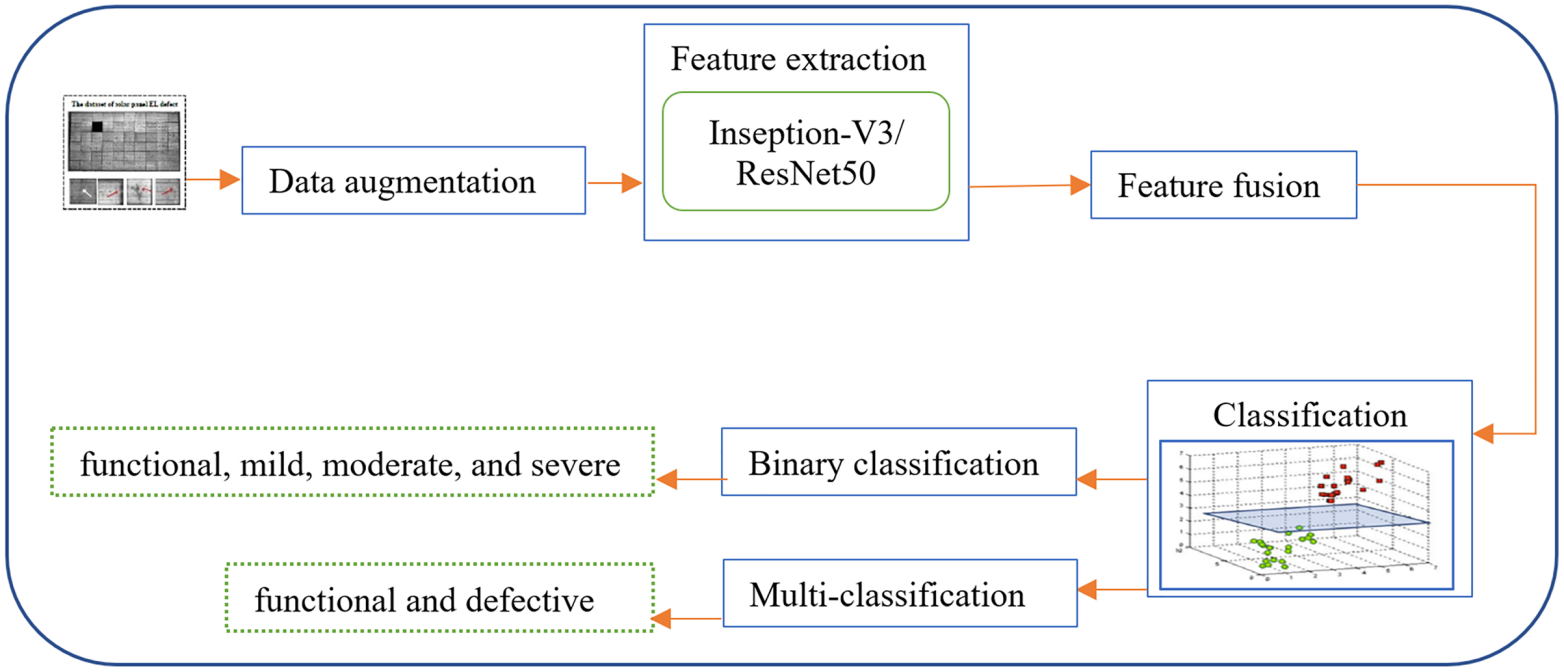
An overview of the proposed hybrid and fully-automated classification system for detecting different types of defects in EL images of solar cells. Figures of cells are licensed under CC BY NC SA 4.0. Figure source credit: (Deitsch et al., 2019).

The proposed system (Fig. 1). depends on fusing the deep feature representations extracted from two different deep learning approaches (Inception-V3 and Resnet50). After applying fusion, the dropout technique is implemented in each training iteration by completely ignoring individual nodes with a probability of 0.5, along with their connections. This method decreases the complex coadaptation of nodes by preventing interdependencies from emerging between them. Finally, a dense layer of a different number of nodes based on the number of predicated classes is added to produce the class label. Herein, the sigmoid activation and Softmax activation function are applied in the binary classification and multi-classification task, respectively.

**Figure 2:**
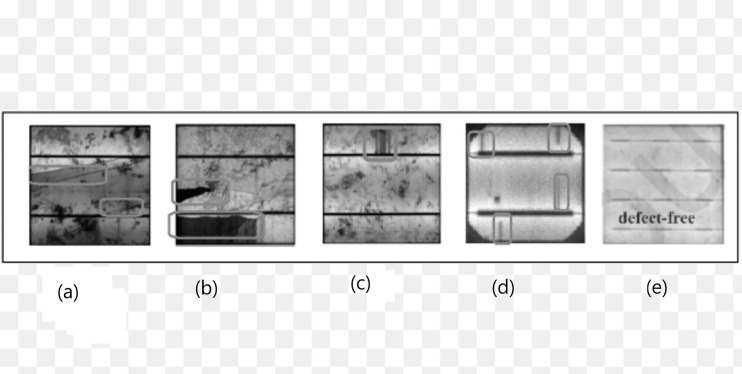
Examples of different defects of PV cells in EL images: (A) different crack pattern (B) broken cell partially (C) interruption of finger (D) shunt fault (E) no-defect. Figures of cells are licensed under CC BY NC SA 4.0. Figure source credit: (Deitsch et al., 2019).

The approaches are tested on an EL image of solar cells with a resolution of (300 × 300) pixels. Examples of employed EL images are shown in Fig. 2. Individual solar cells in PV modules were identified by measuring the median dimensions of the picture regions that correspond to those cells, which are used to calculate the image resolution. Adjustments are necessary to compensate for the fact that the actual image resolution of solar cells in the field may often diverge from the required resolution. Typically, the CNN receptive field equals the minimum image resolution determined by the CNN architecture (*e.g*., the original VGG-19 design utilizes a resolution of (224 × 224) pixels). The image must be up scaled if the resolution is less than the minimal resolution. We took a different approach, encoding this technique directly into the CNN architecture.

### Data acquisition

We used a public collection of monocrystalline and polycrystalline PV modules with high-resolution EL images. At a scale of (300 × 300) pixels, the collection contains 2,624 solar cell images taken from 44 different PV modules, of which 18 are monocrystalline and the other 26 are polycrystalline. The experts initially classified the dataset into four types according to the severity of the problem (Buerhop-Lutz et al., 2018). Classified as the ratio of none defected is 0%, ratio of possibly normal is 33%, the ratio of possibly defective is 66%, and the ratio of defected is 100%. There are 1,508, 295, 106, and 715 images in these categories. Occurrence in monocrystalline and polycrystalline solar modules is inherent and extrinsic faults in the solar cells. Microcracks and cells with electrically isolated and damaged sections, short-circuited cells, open interconnects, and soldering faults are all included in the collection in this manner.

### Data augmentation

To train DNNs, a large dataset is required to achieve high performance in mapping an input to the desired output effectively. Due to the very small size of the training set in the ELPV dataset, different data augmentation techniques can be applied to expand its size. Here, the orientation of the cracks is not important if it is at positive or negative angles. Furthermore, the appearance of cracks at the upper or lower edge does not matter. Thus, different data augmentation techniques can be successfully applied. In this study, several data augmentations have been applied on the training set to enhance the generalization capacity of the adopted deep learning models and avoid overfitting issues. First, all original images have been rotated to 90°, 180° and 270° and added to the original training set to have a different diversity of defect patterns. Second, we flip original images along the x-axis and y-axis. To get more images with a diversity of defect patterns, the Gaussian blur reduce the effect of the dark regions in the EL images and produce new images with different and useful information. Finally, the image contrast technique was also applied to the original EL images to have a wide variety of EL images with different crack appearances.

### Learning deep features representations

Two deep learning networks, Inception-V3 and ResNet50, have been used to extract two set of characteristics from the EL images. The features are then combined to create more accurate data representations. Due to the potential of deep learning feature descriptors to automatically learn the fundamental features in an EL image, their use can significantly reduce the need for handcrafted feature extraction. This is the first work to come up with a way to tell the difference between EL images of working solar cells and those that are malfunctioning. In the end, these feature representations are given to the classifier layer, which assigns them to a specific defect type.

#### Feature extraction using inception-V3 model

Our work with the Inception-v3 architecture is a decent solution between the complexity of the network and the depth of layers that can be implemented (23.8 M parameters). Inception-V3 uses a variety of filter sizes in the same layer. Consequently, the retrieved patterns will be of varied sizes and contain more detailed information. In comparison to AlexNet and VGG, this model network has fewer parameters but is nevertheless capable of learning a more detailed presentation of features. Pre-trained CNN Inception-v3 is the latest and greatest. There are 350 connections between the 316 levels. Three blocks of Inception layers are followed by two grid reduction modules. Global average-pooling and an FC layer are used to combine the final Inception block’s output. Multiple layers of functionality can be gleaned from this architecture. The first input layer is 299 × 299 × 3, and the number of convolution layers is 94 of various filter sizes.

Table 1 provides an explanation of the model that was employed in this work. Following input layer, a scaling layer is proven to be completely placed. At the beginning, activation is done on the first convolution layer, and a weight matrix of dimension 149 × 149 × 32, where (32) is generated as the number of filters. The batch normalization and ReLU activation layers are implemented at the end. The ReLU layer is defined as follows:

**Table 1:** Layers and parameters applied by Inception-V3 model for defective solar cell detection.

Type	Patch size/stride or remarks	Input size
Conv	3 × 3/2	299 × 299 × 3
Conv	3 × 3/1	149 × 149 × 32
Conv padded	3 × 3/1	147 × 147 × 32
Pool	3 × 3/2	147 × 147 × 64
Conv	3 × 3/1	73 × 73 × 64
Conv	3 × 3/2	71 × 71 × 80
Conv	3 × 3/1	35 × 35 × 192
3 × inception	1 × 1 and 3 × 3/1	35 × 35 × 288
5 × inception	n × 1, 1 × n, and n × n/1	17 × 17 × 768
2 × inception	1 × 1, 1 × 3, 3 × 1, and 3 × 3/2	8 × 8 × 1,280
Pool	8 × 8	8 × 8 × 2,048
Linear	Logits	1 × 1 × 1,280
Softmax	Classifier	1 × 1 × 1,000

(1) $$Re_i^{\left( l \right)} = \max \left( {hv,hv_i^{\left( {l - 1} \right)}} \right)$$

A pooling layer is inserted between them to obtain neurons that are active. It is 2 × 2 in the initial max-pooling layer. The max-pooling can be described mathematically as follows:

(2) $$mx_1^{\left( q \right)} = {\rm \; }mx_1^{q - 1}$$

(3) $$mx_2^{\left( q \right)} = {\rm \; }\displaystyle{{mx_2^{\left( {q - 1} \right)} - F\left( q \right)} \over {{S^q}}} + 1$$

(4) $$mx_3^{\left( q \right)} = {\rm \; }\displaystyle{{mx_3^{\left( {q - 1} \right)} - F\left( q \right)} \over {{S^q}}} + 1$$

where the stride is represented by ${S^M}$, ${\rm \; }mx_1^M,{\rm \; }mx_2^M,{\rm \; }{\rm and}{\rm \; }mx_2^M{\rm \; }$ Filters for feature set maps *e.g*. 2 × 2 and 3 × 3 have been defined. Additional layers including addition and concatenation are also included in this architecture. Finally, an average based on pooling layer has been added. As a result of the activation, a features map with dimensions of 1 × 1 × 2,048 is created as a weight matrix. Learnable weights are 1,000 × 2,048 for FC, while the feature matrix is 1 × 1 × 1,000 for the final layer. The FC layer is represented mathematically as follows:

(5) $$Fc_i^{\left( l \right)} = {\rm \; }f\left( {z_i^{\left( l \right)}} \right)with{\rm \; }z_i^{\left( l \right)} = {\rm \; }\mathop \sum \limits_{j = 1}^{n{1^{\left( {l - 1} \right)}}} \mathop \sum \limits_{r = 1}^{n{2^{\left( {l - 1} \right)}}} \mathop \sum \limits_{s = 1}^{n{3^{\left( {l - 1} \right)}}} w_{i,j,r,s}^{\left( l \right)}{\left( {Fc_i^{\left( {l - 1} \right)}} \right)_{r,s}}$$

#### Feature extraction using ResNet50 model

**Figure 3:**
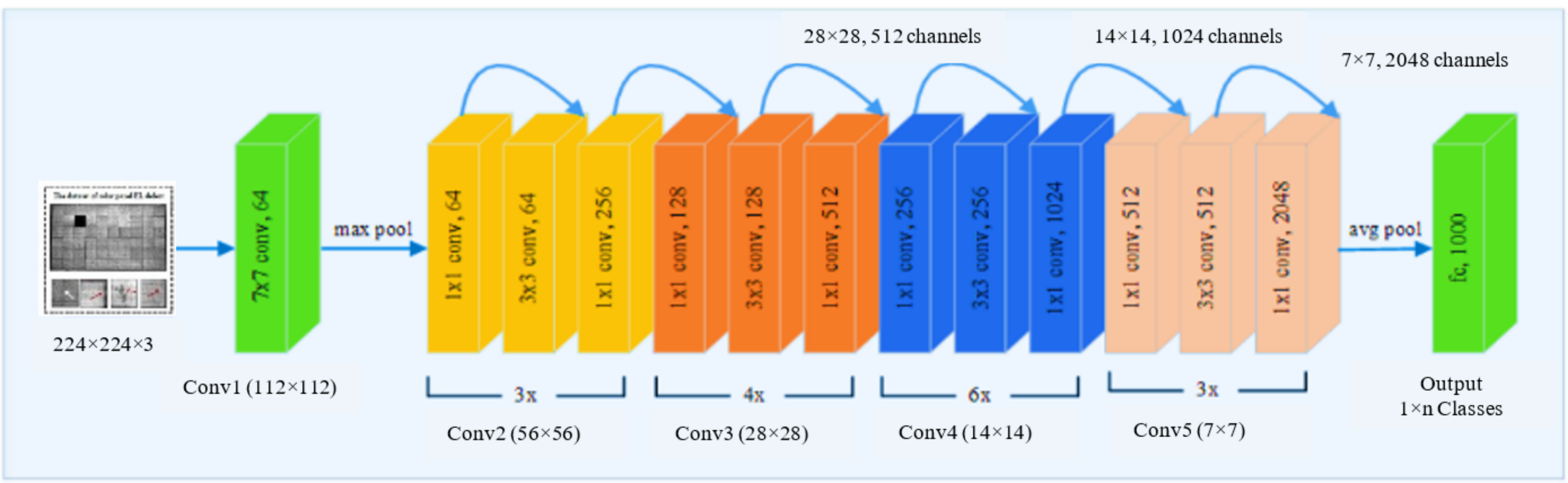
The pre-trained ResNet50 structure employed in the feature extraction stage. Figures of cells are licensed under CC BY NC SA 4.0. Figure source credit: (Deitsch et al., 2019).

ResNet is one of the most advanced deep models for image categorization. When the deep network cannot be trained, ResNet overcome the challenge to ameliorate classification accuracy while simultaneously reducing the number of parameters. Therefore, we use ResNet50 as a deep convolutional feature extractor. ResNet adds to the network’s depth without affecting classification performance based on residual learning. The remainder modules have two paths: one executes two or three convolutions on the input feature to produce the residual of that feature, and the other is a direct path from the input feature. The output of the residual module is the sum of the outputs of these two pathways. As shown in Fig. 3, ResNet50 was used to extract features. The weights that have been pre-trained on EL datasets are used to initialize the network. Deeper layers’ learnt weights tend to be more class-specific, such as ResNet50’s completely connected layer. We were interested to know how well the output vectors of the previous convolutional layers could be classified. Networks with deep convolutional layers are significant features when applied correctly. We used the last residual unit outputs from convolutional layers 3, 4, and 5 as feature vectors. The third layer features have a smaller dimension than the fifth layer features. Table 2 shows that we used ResNet50 in our investigation.

**Table 2:** Layers and parameters applied by resnet50 model for defective solar cell detection.

Layer name	Output size	50-layer
Conv1	112 × 112	7 × 7, 64, stride 2
Conv2-x	56 × 56	3 × 3 max pool, stride 2
		$\left[ {\; \matrix{ {1\; \times {\rm \; }1,{\rm \; }64} \cr {3\; \times {\rm \; }3,{\rm \; }64} \cr {1\; \times {\rm \; }1,{\rm \; }256} \cr } } \right]$×3
Conv3-x	28 × 28	$\left[ {\; \matrix{ {1\; \times {\rm \; }1,{\rm \; }128} \cr {3\; \times {\rm \; }3,{\rm \; }128} \cr {1\; \times {\rm \; }1,{\rm \; }512} \cr } } \right]$×4
Conv4-x	14 × 14	$\left[ {\matrix{ {1\; \times {\rm \; }1,{\rm \; }256} \cr {3\; \times {\rm \; }3,{\rm \; }256} \cr {1\; \times {\rm \; }1,{\rm \; }1,\!024} \cr } } \right]$×6
Conv5-x	7 × 7	$\left[ {\matrix{ {1\; \times {\rm \; }1,{\rm \; }512} \cr {3\; \times {\rm \; }3,{\rm \; }512} \cr {1\; \times {\rm \; }1,{\rm \; }2,\!048} \cr } } \right]$×3
	1 × 1	Average pool, 1,000-d fc, softmax
FLOPs		1.8 × 10^9^

**Figure 4:**
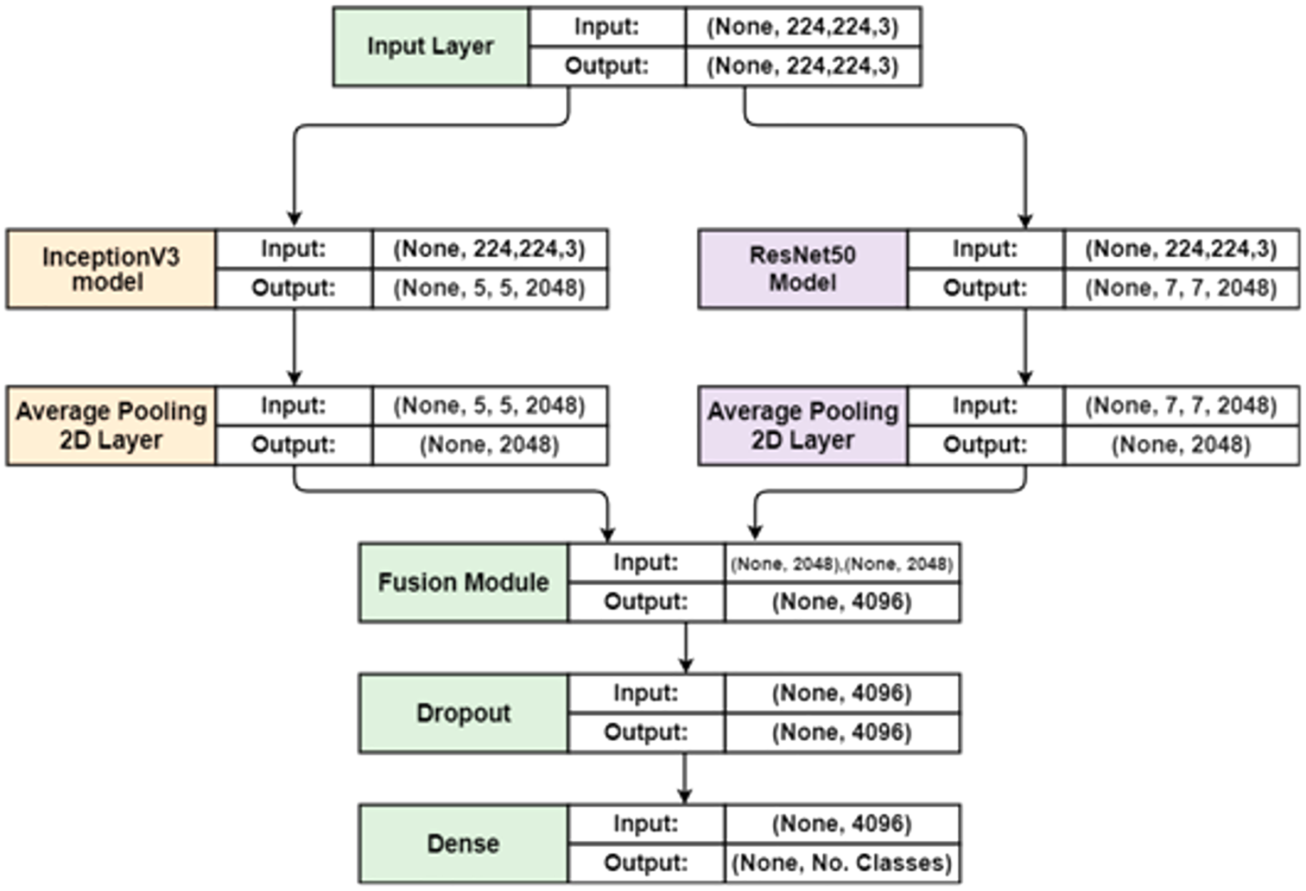
The representation of the proposed hybrid deep feature fusion.

To improve the classification accuracy, a more robust feature vector is obtained using feature fusion process. The most recent research shows that the fusion process enhances overall performance, however the major drawback is that it requires a lot of time to compute. Although our primary goal is to enhance classification accuracy. New Parallel Maximum Covariance (PMC) features fusion method is used to achieve this goal. The lengths of the two feature extraction vectors must be equalized in this method. We then calculate the single-matrix fusion’s greatest covariance. The proposed feature fusion approach is depicted in Fig. 4.

Consider ${{\bf \varphi }^{\left( {{\bf k}1} \right)}}$ and ${{\bf \varphi }^{\left( {{\bf k}2} \right)}}$ are two different feature vectors based deep learning of two different dimensions ***(n × m)*** and ***(n × q)***. The number of images is represented by ***n, m*** is the Inception-V3 deep learning feature vector of (***n* × 2,048**) dimensions, and the ResNet50 feature vector of **(*n* × 2,048)** dimensions represents by ***q***. To make equalization between lengths of feature vectors, we need to know what the maximum length of a certain vector after that calculate the padding of average value. The average feature is computed from vector with a higher vector. Assume ***n*** is a random column m unit vector displaying some pattern in the ${\bf \varphi }1$ field. The pattern in the field of ${\bf \varphi }2$ is represented by a random unit of column vector which indicated by **b**. The projections of time series on row vectors are formulated in the following manner:

(6) $${x_1} = {\rm \; }\varphi _1^T{\varphi ^{\left( {k1} \right)}}$$

(7) $${x_2} = {\rm \; }\varphi _2^T{\varphi ^{\left( {k2} \right)}}$$

For optimal solution ${\varphi }1$ and ${\varphi }2$ maximize their covariance as follows:

(8) $${c^\sim} = {\rm \; }Cov{\rm \; }\left[ {{\rm \; }{x_1},{\rm \; \; }{x_2}} \right]$$

(9) $${c^\sim} = {\rm \; }Cov{\rm \; }\left[ {\varphi _1^T{\varphi ^{\left( {k1} \right)}},\varphi _2^T{\varphi ^{\left( {k2} \right)}}} \right]$$

(10) $${c^\sim} = {\rm \; }\displaystyle{1 \over {n - 1}}\left[ {\varphi _1^T{\varphi ^{\left( {k1} \right)}}\left( {\varphi _2^T{\varphi ^{\left( {k2} \right)}}} \right)} \right]$$

(11) $${c^\sim} = \varphi 1\left( {{C_{\varphi 1\varphi 2}}} \right)\varphi 2$$

(12) $${C_{\varphi 1\varphi 2}} = {\rm \; }\displaystyle{1 \over {n - 1}}\left[ {{\varphi ^{\left( {k1} \right)}}{\varphi ^{{{\left( {k2} \right)}^T}}}} \right]$$

${{C}_{{\varphi }1{\varphi }2}}{\rm \; }$is the covariance value between ${\varphi }1$ and ${\varphi }1$ whose ***i***^***th***^ and ***j***^***th***^ features are ${\varphi }{\bf i}\left( {\bf t} \right)$ and ${\varphi }{\bf j}\left( {\bf t} \right)$.

The last fused vector saves the feature pair ***i*** and ***j*** with the highest covariance ${{C}_{{\varphi }1{\varphi }2}}$. Once all pairs have been compared, the procedure repeats again. Finally, a fused vector is acquired, represented by ${{\bf \varphi }^{\left( {{\bf fu}} \right)}}$ of dimensions $\left( {{N\; } \times {\; K}} \right),$ where ${K}$ specifies the feature length, which changes depending on the features picked. (***N* × 4,096**) is the feature length in this study. Dropout is a method for avoiding overfitting neural networks by removing units at random. We set a dropout of **0.5** for the fully connected layer in our investigation.

### The proposed training strategy

This work pre-trained the weight configurations of ResNet50 and Inception-V3 architectures. Initializing entire weights with the weight configurations has been performed of the pre-trained architectures in the learning strategy. Then, Adam optimizer is employed to optimize them through the current task. To detect most relevant and significant features the pre-trained models based on their weight configurations have been learned. Training time could be reduced, the ability of model generalization could be enhanced, and over-fitting can be prevented. In the first classification scenario we have performed binary classification to classify defective solar cell into functional and defective categories. However, multi classification scenario has been performed to classify defective solar cells into four different categories namely, severe, moderate, functional, and mild. Both previous scenarios are performed based on unaugmented and augmented dataset. During the process of learning, the proposed deep feature representation model has been trained based on distributing dataset into 80% as training data and 20% as testing data. The selection dataset process was done randomly. Finally, the performance of training process as well as the final performance based on using proposed deep hybrid feature have been reported.

### Image classification stage

Combining the findings of several models, the ensemble of deep CNNs may prove to be an effective strategy for improving outcomes. The procedure of classification involves the organized and methodical assignment of every structure to one and just one category in a system of classes that are mutually exclusive and do not overlap categories. In this study, binary and multi-classes classification are carried out using sigmoid and Softmax activation layers, respectively. The fault probability class of a solar cell was determined utilizing binary class classification technique in this work. Extracted features based deep network models can be used to identify functional and faulty cells in the EL images of this data set. The class 0 (functional) samples make up 65% of the EL dataset, while class 1 samples make up 35% (defective). For binary classification of EL images, sigmoid activation is employed. In this work, the parameters of the adopted deep learning models are trained using the Adam optimizer algorithm for 300 epochs with a mini-batch size of 16, a learning rate set to be 0.01, a weight decay value of 0.0005, and a momentum value of 0.95. In classification of EL images based, the first dense layer has 64 neurons instead of 128.

## Experimental results

Experiments for performance evaluation conducted with the final model are presented in this section and compare our own findings with the results of recently developed approaches. This section involves three evaluation subsections to show the affective of each stage that has been used in the proposed model. First, the result of the data augmentation has been introduced in the first subsection to show the affective of the augmenter results compared to the original data. Second, test the proposed binary and multi-classification model and the results achieved from different experiments are then discussed. Finally, to highlight the powerful of the proposed hybrid model, our own findings have been compared with most resent affective approaches. For additional validation, the proposed system is tested on the publicly available EL image dataset (ELPV dataset), which comprises 2,624 high-resolution EL image-extracted images of solar cells (Buerhop-Lutz et al., 2018).

To benefit from the described dataset for training and testing stages, the dataset has been divided into two groups, 80% training and 20% testing as illustrated in Table 3. The running time of the adopted deep learning models was measured by executing them on a PC with the Windows 10 operating system, an Intel(R) Core(TM) i7-4790 CPU, and 32 GB of RAM. The training time for all the conducted experiments was ranging from 30–45 min for both the Inception-V3 and ResNet50 models. On the other hand, the test time per image from the image input until the final decision is about 1.01 and 1.14 ms for the Inception-V3 and ResNet50 models, respectively.

**Table 3:** Distribution of the training and testing data set.

Class	No. of images	Normal	Mild	Moderate	Severe
**Training**	2,100	577	480	499	544
**Testing**	524	144	120	124	136

### Impact of data augmentation on results

Train the deep learning models on small datasets is a significant challenge due to the overfitting problem that became as a significant issue in this situation. Data augmentation procedures are implemented on original images to obtain more information that can help to train the deep learning model. For data augmentation, we have performed rotation, flipping, and cropping operations. It was necessary to carefully examine the newly obtained images to generate new set of images with the same labels, to identify variations from the original images, and to extract important information from them. Data augmentation procedure results in an improvement in model accuracy, leading to the conclusion that the data augmentation operations that were chosen are successfully implemented. They help to reduce the difference between images and aid in the model’s learning of more representative feature subsets, which results in a significant improvement in recognition accuracy. Experiments were carried out with Inception-V3, ResNet50, and proposed models with various data augmentation settings to determine the effect of data augmentation. All features from Inception-V3, ResNet50m and the proposed model are extracted for the purposes of these experiments, and the dataset is separated into two different parts: 80% and 20% as training and testing, respectively. From results obtained in Tables 4, 5, 6, and 7, the highest accuracy is achieved with data augmentation techniques.

**Table 4:** Performance comparison to assess the efficiency of the adopted models without applying data augmentation in the binary classification task.

Method	Dimension	Accuracy (%)	Recall (%)	Precision (%)	F1-Score (%)	Specificity (%)
Inception-V3	2,048	47.41	44.44	47.24	45.80	50.37
ResNet50	2,048	55.19	52.59	55.47	53.99	57.78
Hybrid model	4,096	60	60.74	59.85	60.29	59.26

**Table 5:** Performance comparison to assess the efficiency of the adopted models with the data augmentation in the binary classification task.

Method	Dimension	Accuracy (%)	Recall (%)	Precision (%)	F1-Score (%)	Specificity (%)
Inception-V3	2,048	94.44	94.81	94.12	94.46	94.07
ResNet50	2,048	95.91	96.30	95.59	95.94	95.52
Hybrid model	4,096	98.15	98.51	97.78	98.14	97.79

**Table 6:** Performance comparison to assess the efficiency of the adopted models without applying the data augmentation in the multi-classes classification task.

Method	Dimension	Accuracy (%)	Recall (%)	Precision (%)	F1-Score (%)	Specificity (%)
**Inception-V3**	2,048	48.39	48.5	52.5	48.03	82.5
**ResNet50**	2,048	52.68	52.75	55.5	52.51	84
**Hybrid model**	4,096	61.15	61.75	66.02	61.03	87.25

**Table 7:** Performance comparison to assess the efficiency of the adopted models with applying the data augmentation in the multi-classes classification task.

Method	Dimension	Accuracy (%)	Recall (%)	Precision (%)	F1-Score (%)	Specificity (%)
**Inception-V3**	2,048	87.67	87.01	87.05	86.75	95.75
**ResNet50**	2,048	91.60	91.61	91.55	91.58	91.52
**Hybrid model**	4,096	95.35	95.50	95.53	95.25	98.50

### Image classification results

Two experiments have been implemented to evaluate the outcome of the proposed model. First, the PV cells divided into two classes (normal and defective) using the ground truth labels to train and test the proposed hybrid model. Second, the same dataset divided into four classes (normal, mild, moderate, and severe) and using them to train a new model that can give more specific classification for the testing data. As a result, this study presents two-class classification results, one for normal classes and another for the defective classes. EL images of normal cells show that their surfaces are homogeneous and that the faults in those cells are distinct from the background in the image. Cells in these classes have a wide range of textures, and defects in those cells are frequently similar in appearance to the background in an EL image, making it difficult to distinguish between them. Both classifications were evaluated for their performance and the results of this evaluation are presented in the following subsection.

**Figure 5:**
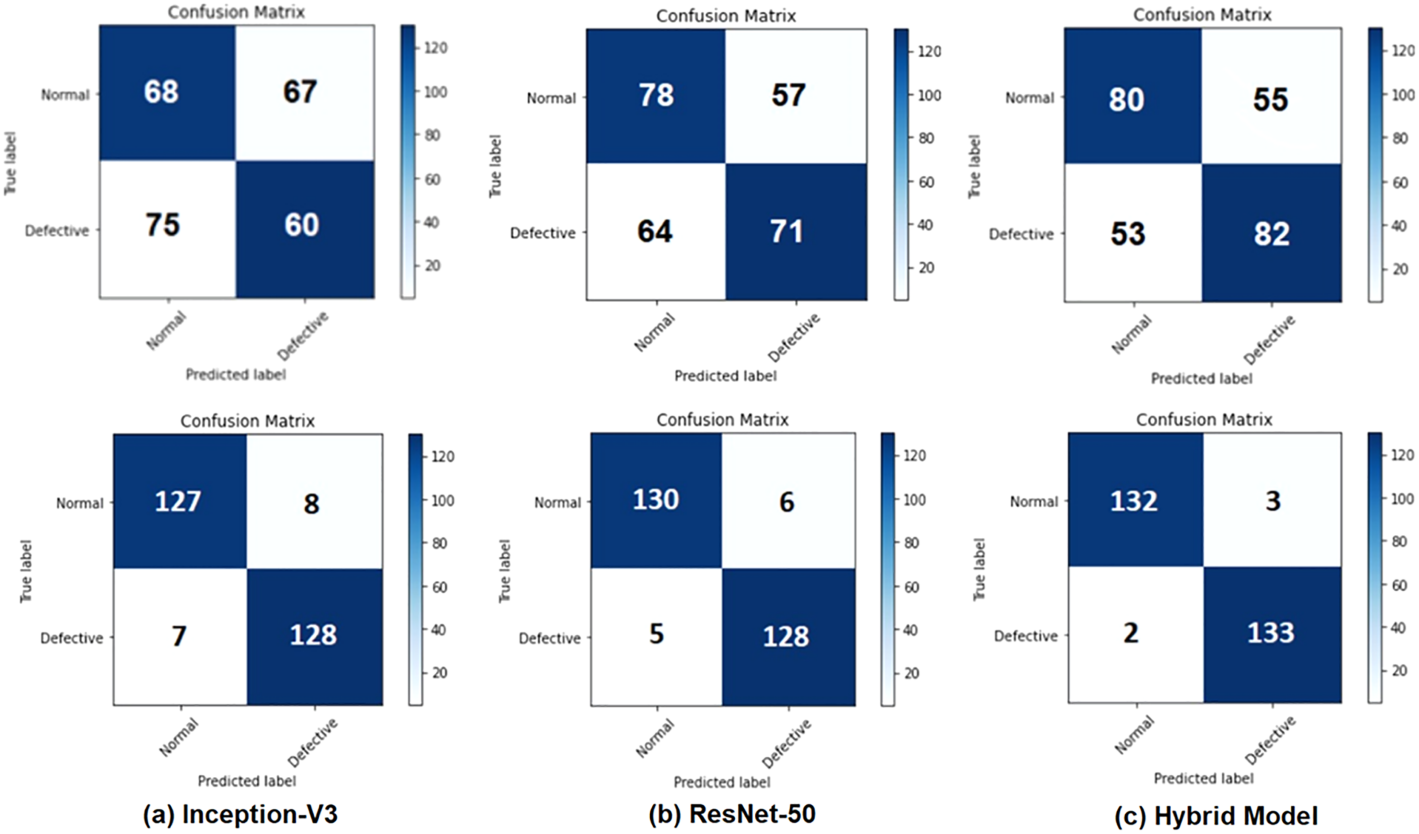
Confusion matrices for the binary classification task: (A) Inception-V3 model, (B) ResNet50 model, and (C) the hybrid system (top row) before applying the data augmentation and (bottom row) after applying the data augmentation.

Tables 5 and 6 demonstrate results of evaluation of performance of test before applying data augmentation as well as after applying data augmentation. features extracted from Inception-V3, ResNet50, and integrated features are presented in Tables 5 and 6. Accuracy rates were obtained as 94.44% for Inception-V3, 95.91 for ResNet50 and 98.15% for combined features. features extracted from Inception-V3 network achieved worst result between m. proposed model (combined features) provided better results than or models when used independently. Confusion matrices are given in Fig. 5 for binary classification task before and after data augmentation.

**Figure 6:**
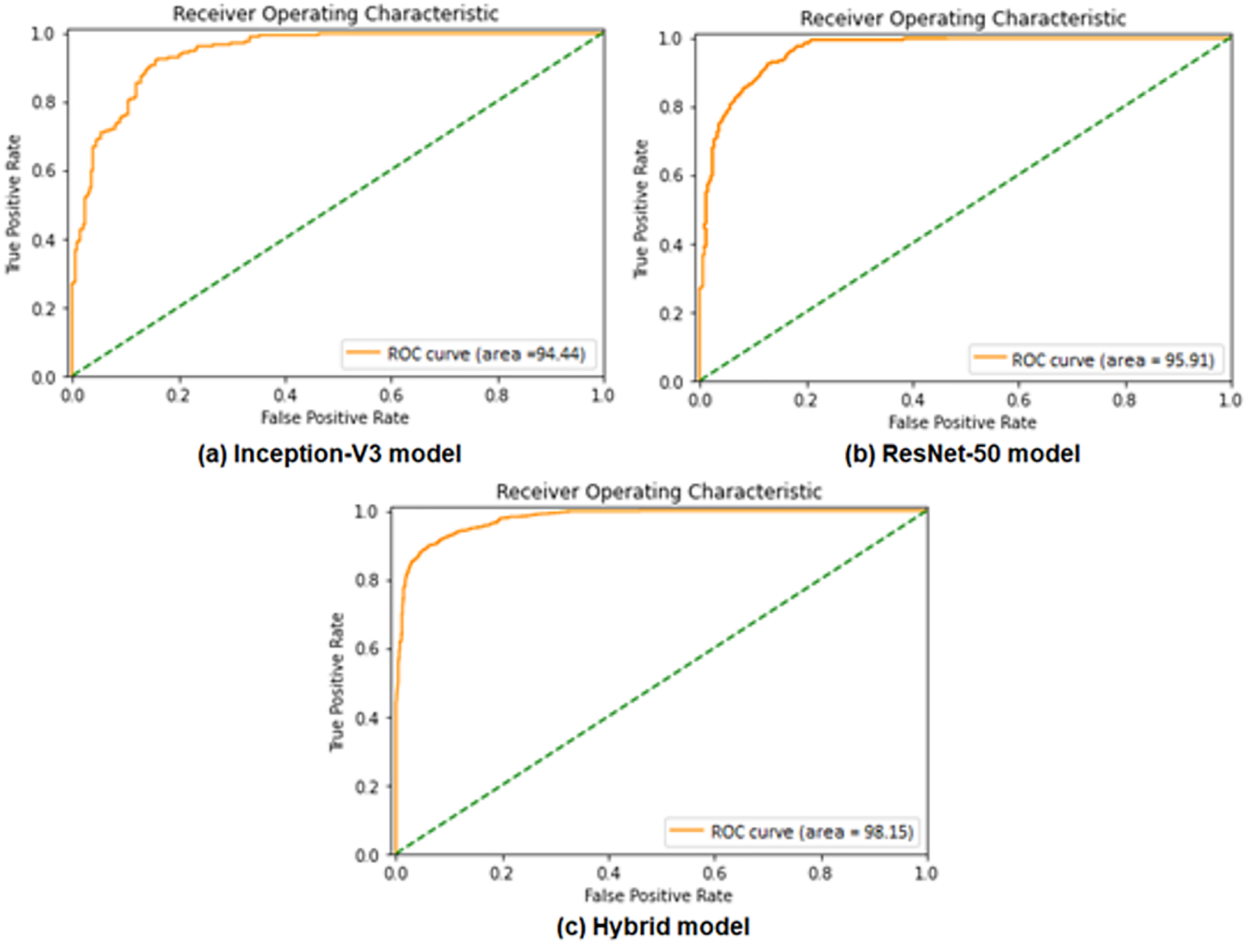
Comparisons of the ROC curves for the adopted deep learning models in the binary classification task: (A) Inception-V3, (B) ResNet50, and (C) Hybrid model.

Performance evaluation is demonstrated using ROC curves, which represent the performance of binary classification based on the sigmoid activation function at different false positive rates. ROC curve in Figs. 6A and 6B presents the performance result of the Inception-V3 and ResNet50 models, respectively. It shows that the two models achieved ROC curve performance nearly similar by obtaining 94.44% for Inception-V3 model and 95.91% for ResNet50 model. Figure 6C shows the ROC performance for the proposed hybrid model by obtaining 98.15%. ROC of hybrid Inception-V3 and ResNet50 performs results outperformed of each model independently.

#### Multi-classes classification task

A four-class classification performance experiments utilizing the Softmax activation function are presented as shown in Tables 7 and 8. The integrated feature vectors in four class classification tasks have achieved the best results. The accuracy rates of 48.39% and 87.67% for Inception-V3, 52.68% and 91.60% for ResNet50, finally 98.15% and 95.35% for combined features were achieved for each model before and after applying data augmentation, respectively.

**Table 8:** Comparative performance (%) for binary defective solar cells classification approaches.

Approaches	Feature dimension	Image size	Accuracy	Recall	Precision	F1-Score	Specificity
**L-CNN** (3)	2,000	100 × 100	89.33	95.42	90.44	92.86	–
**DFB-SVM** (3)	2,000	100 × 100	94.52	97.36	94.79	96.06	–
**Deep Siamese CNN** (12)	4,096	100 × 100	93.02	93	93	92.49	–
**VGG + Linear SVM** (5)	4,096	300 × 300	82.44	–	–	82.52	–
**CNN** (5)	4,096	300 × 300	88.42	–	–	88.39	–
**Light CNN** (7)	4,096	100 × 100	93.02	93	93	92.49	–
**MVGG19** (29)	2,500	300 × 300	97.88	98.57	96.49	97.5	–
**Inception-V3**	2,048	300 × 300	94.44	94.81	94.12	94.46	94.07
**ResNet50**	2,048	300 × 300	95.91	96.3	95.59	95.94	95.52
**Hybrid Model**	4,096	300 × 300	98.15	98.51	97.78	98.14	97.79

**Figure 7:**
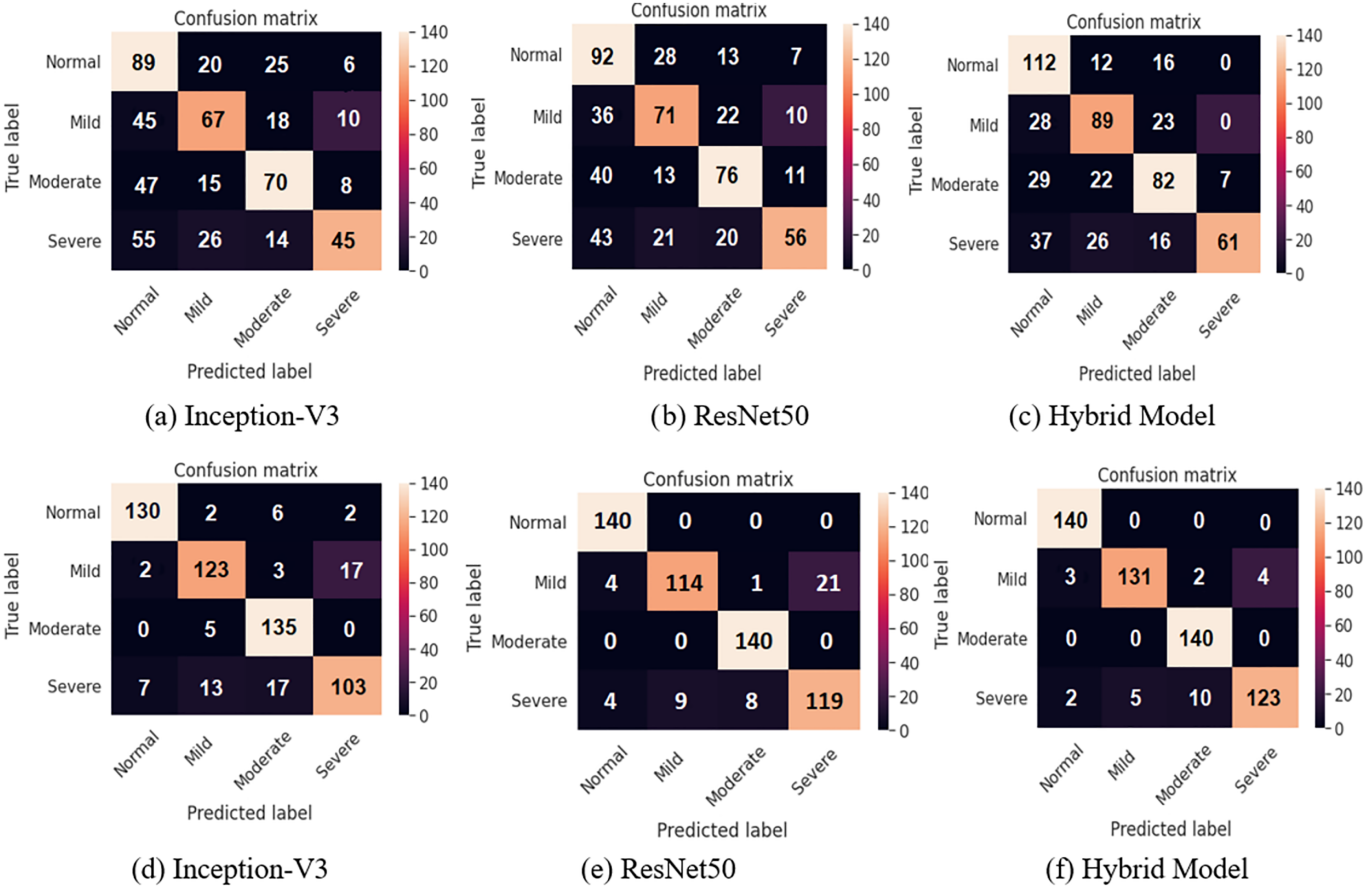
Confusion matrices for two class classification using sigmoid classifier (A, B, and C) before applying the proposed data augmentation procedure, and (D, E, and F) after applying the proposed data augmentation procedure.

Figure 7 shows the confusion matrices of all the methods before and after applying data augmentation. For the two class confusion matrices, the proposed model obviously outperformed both Inception-V3 and ResNet50 for all classes. Mild and Severe cell images with defects such as the background have a high likelihood of being misclassified. Cell images with minor defects are incorrectly classified in the case of the Normal and Moderate images. Misclassification of two-class classification tasks occurs due such EL images of the cells are at the boundary between obviously differentiable normal and defective cells, resulting in incorrect classification.

### Comparison study

Using the same dataset that we used in our study, we compare the results of recently developed approaches with our own findings. Table 8 shows the results of the study compared to previous studies. Compared to other techniques, our proposed model is superior with 98.15% accuracy, 98.51% recall, 97.78% precision, and 98.14% F1 score.

Performance of the proposed system was also compared with most current state-of-the-art existing works in term of multi-classification task, as shown in Table 9. Our method achieved 95.35% accuracy, 95.5% recall, 95.53% precision, and 95.25% F-Score. The proposed approach outperformed all prior ones in terms of accuracy and other metrics, as can be shown in the results.

**Table 9:** Comparative performance (%) for multi-defective solar cells classification approaches.

Approaches	Feature dimension	Image size	Accuracy	Recall	Precision	F1-score	Specificity
**L-CNN** (3)	2,500	100 × 100	82.58	91.58	80.71	85.80	–
**DFB-SVM** (3)	2,500	100 × 100	89.63	96.59	87.01	91.55	–
**L-CNN** (3)	2,048	300 × 300	82.44	–	–	82.52	–
**DFB-SVM** (3)	2,048	300 × 300	88.42	–	–	88.39	–
**Siamese** (12)	–	130 × 130	74.75	73.75	74.75	73.75	–
**SURF-SVM** (30)	–	100 × 100	72.74	–	–	–	–
**CNN** (30)	–	100 × 100	91.58	91.85	91.57	91.57	–
**Inception-V3**	2,048	300 × 300	87.67	87.01	87.05	86.75	95.75
**ResNet50**	2,048	300 × 300	91.60	91.61	91.55	91.58	91.52
**Hybrid Model**	4,096	300 × 300	95.35	95.50	95.53	95.25	98.50

The classification of solar cell faults in EL images is a challenging process because of the intrinsic silicon structure that creates crystal grain boundaries in solar cells. This makes the classification of solar cell defects particularly difficult. It becomes more difficult to distinguish between defective and normal areas because there is a lack of a large enough collection of both normal and defective cell images, as well as normal, mild, moderate, and severe cell images, is another important consideration. At a certain level, these issues are addressed by data augmentation.

Research limitations should be highlighted. First, the EL images suffer from a specific type of noise. Further investigation is required to understand and reduce the noise and keep the important texture information. Second, the proposed hybrid model needs to be trained and tested on more data from different environments to have more challenges with different data. Finally, the similarity between the texture of the normal cells and defected cells is a hard challenge. It still needs more investigation to find more effective descriptor that can reduce overlap between classes.

## Conclusions and future work

The experimental results have demonstrated the efficiency of the developed framework in accurately and correctly identifying the defective solar cells in the EL images by achieving an accuracy rate of 98.15% and 95.35% in binary classification and multi-classes classification tasks, respectively. Clearly, further experiments will be required using a larger and more challenging EL images dataset to prove the reliability of the proposed defective solar cell identification framework. In addition to investigating the potential of training the adopted deep learning models on top of pre-processed EL images using some of the well-known image enhancement techniques (*e.g*., histogram equalization, linear contrast adjustment, etc.). We might be able to guide the learning process of the adopted deep learning models to learn more distinct feature representations instead of using the raw EL image data. Finally, some of the uncertainty quantification methods can be employed to reduce the effect of uncertainties during both learning and decision-making processes.

## Additional Information and Declarations

### Data Availability

The following information was supplied regarding data availability: The data is available at GitHub: https://github.com/zae-bayern/elpv-dataset. The code is available at GitHub: https://github.com/Dilovan1/Defective-solar-cell.
